# Extracellular vesicle lipidomics liquid biopsy reveals systemic and tumor microenvironment metabolic reprogramming in ovarian cancer

**DOI:** 10.1002/1878-0261.70327

**Published:** 2026-10-07

**Authors:** Shikha Rani, Dominic Guanzon, Andrew Lai, Katherin Scholz‐Romero, Sarah Reed, Edita Ritmejeryte, Rohan Lourie, Naven Chetty, Kaltin Ferguson, Carlos Salomon

**Affiliations:** ^1^ Translational Extracellular Vesicles in Obstetrics and Gynae‐Oncology Group, Frazer Institute, Faculty of Health, Medicine and Behavioural Sciences, Royal Brisbane and Women's Hospital The University of Queensland Herston Australia; ^2^ UQ Centre for Extracellular Vesicle Nanomedicine, Faculty of Health, Medicine and Behavioural Sciences The University of Queensland Herston Australia; ^3^ Mass Spectrometry Facility, Frazer Institute University of Queensland Brisbane Australia; ^4^ Mater Research Institute University of Queensland, Translational Research Institute Woolloongabba Australia; ^5^ Mater Health Services South Brisbane Australia

**Keywords:** biofluids, extracellular vesicles, lipidomics, liquid biopsy, ovarian cancer cells

## Abstract

Ovarian cancer is the most lethal gynecological malignancy, due to late‐stage diagnosis, tumor heterogeneity, and lack of biomarkers for early detection. Extracellular vesicles (EVs) in plasma and ascites offer a minimally invasive source of tumor‐derived information, yet their lipid composition and relationship to tumor heterogeneity remain poorly defined. We performed lipidomic profiling of EVs from plasma and ascites of high‐grade serous ovarian cancer patients and from ovarian cancer and control cell lines to identify signatures of tumor progression and microenvironmental interactions. A lipid panel enriched in ovarian cancer EVs was evaluated in EVs and plasma across two independent cohorts (*n* = 146). Combined with CA125, this panel improved detection, particularly against benign adnexal masses, achieving an AUROC of 0.99 in the verification cohort in both formats. Ovarian cancer EVs preferentially accumulated triglycerides, including ether‐linked species, whereas parental cells were enriched in phospholipids and ceramides. EV lipid signatures reflected tumor‐associated metabolic reprogramming and captured tumor heterogeneity across cell models. These findings show that EV lipidomics from accessible biofluids offers a non‐invasive window into ovarian cancer metabolism and candidate biomarkers for patient stratification and monitoring.

## Graphical abstract

Extracellular vesicle (EV) lipidomic profiling of plasma and ascites from patients with high‐grade serous ovarian cancer and of ovarian cancer cell lines reveals enrichment of triglycerides (TGs), diacylglycerols (DGs), phosphatidylcholine (PCs), sphingomyelins (SMs), reflecting tumor metabolic reprogramming. EV lipid signatures capture systemic and microenvironmental heterogeneity and provide candidate biomarkers for non‐invasive disease stratification. Created in biorender. Rani, S. (2026) https://BioRender.com/p1rbh56.

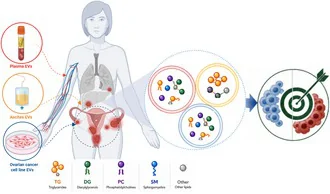

**Abbreviations**
BA: bile acid
CAR: acylcarnitine
CDP‐DAG: cytidine diphosphate diacylglycerol
CE: cholesteryl Ester
Cer: ceramide
CerP: ceramide‐phosphate
CerPE: ceramide phosphoethanolamine
CL: cardiolipin (or diphosphatidylglycerol)
DG or DAG: diacylglycerol
DGDG: digalactosyl DG
DGMG: digalactosyl MG
DGPP: DG Pyrophosphate
DMPE: dimethyl PE
EOC: epithelial ovarian cancer
EVs: extracellular vesicles
FA: fatty acid
GlcdE: glycidol Ester
GM2: monosialoganglioside GalNAc
Hex2Cer: dihexosylceramide
Hex3Cer: trihexosylceramide
HexCer: hexosylceramide
HGSOC: high‐grade serous ovarian cancer
LPC: lysophosphatidylcholine
LPG: lysophosphatidylglycerol
LPS: lysophosphatidylserine
MG or MAG: monoacylglyceride
MGDG: monogalactosyldiacylglycerol
MGMG: monogalactosyl MAG
MMPE: monomethyl PE
NAPE: N‐acyl PE
NTA: nanoparticle tracking analysis
OAHFA: O‐acyl‐(ω‐hydroxy) fatty acid
PA: phosphatidic acid
PC: phosphatidylcholine
PE: phosphatidylethanolamine
PG: phosphatidylglycerol
PI: phosphatidylinositol
PIP: PI phosphate
PIP2: PI biphosphate
PIP3: PI triphosphate
PS: phosphatidylserine
SE: steryl ester
SHexCer: sulfohexosylceramide
SM: sphingomyelin
SPE: solid‐phase extraction
TG or TAG: triacylglycerol/triglyceride

## 1 Introduction

Ovarian cancer (OC) remains the primary cause of gynecological cancer‐related deaths worldwide. Among its subtypes, high‐grade serous ovarian cancer (HGSOC) is the most common and aggressive, contributing approximately 70% of ovarian cancer cases and the majority of the cancer‐related deaths in women. The poor prognosis of HGSOC is largely driven by peritoneal spread or metastasis, eventual recurrence and the development of chemoresistance which together contribute to 80–90% of all ovarian cancer deaths [1, 2, 3]. Epithelial ovarian cancer is a highly heterogeneous disease, causing a significant challenge for the biomarker discovery. It is categorized into five main histological subtypes: high‐grade serous, low‐grade serous, endometrioid, mucinous and clear‐cell carcinoma [4]. Mutations in key genes including TP53, PTEN, KRAS and Rb1 are major oncogenic drivers implicated in the initiation and progression of ovarian tumors [5, 6, 7]. In particular, HGSOC is characterized by almost universal inactivating mutations in the TP53 tumor suppressor gene accompanied by genomic alterations that contribute to intratumoral heterogeneity. Beyond its classical role as a tumor suppressor, TP53 influences cellular metabolic pathways [8], and the mutant TP53 variants have been shown to regulate glucose metabolism and modulate fatty acid synthase (FASN), a critical enzyme involved in lipid biosynthesis [9, 10]. Moreover, TP53 mutation leads to changes in the phosphatidylinositol acyl chain composition [11]. Such remodeling introduces a lipid‐mediated regulatory mechanism influencing the PI3K/AKT signaling pathway. In addition, enhanced lipogenesis and lipid uptake by ovarian cancer cells promote chemoresistance and dampen the adaptive immune responses, thereby facilitating tumor survival and progression [12].

Extracellular vesicles (EVs) are nano‐sized vesicles typically of size 50–150 nm in diameter released by a wide variety of cells via the endosomal system [13]. EVs are known to circulate throughout the bloodstream and are recently identified as crucial mediators of intercellular communication and increasingly recognized for their role in tumor progression and metastasis, making them a promising tool for disease biomarker discovery [14, 15]. Among the diverse bioactive cargoes in EVs, the membrane lipids are particularly abundant; however, they remain less explored compared to other cargoes such as proteins and nucleic acids [16, 17]. Despite the inherent heterogeneity of EVs, specific EV membrane lipid profiles allow for the identification and analysis of EVs originating from cancer cells, uniquely connecting lipidomics to oncogenic processes [16, 18, 19]. However, rigorous assessment for EV lipidomics remains a significant challenge in the field due to the complexity of the EV population and technical limitations, resulting in a persistent lack of disease‐specific lipid signatures.

Cellular processes such as proliferation, inhibition of apoptosis, and acquiring the invasive capacity are the distinctive features of tumor development and progression. These processes are driven by significant functional and morphological alterations at the cell membrane level [20]. As lipids constitute at least 50% of the cellular membrane composition [21], they not only provide essential structural integrity to the EVs but also act as critical signaling molecules [22, 23]. The alterations in the lipid environment can modulate the membrane fluidity, which in turn affects the function of intrinsic membrane proteins and downstream cell signaling pathways [24], while lipid composition plays key roles in the stability of EVs and clearance of microparticles from the circulation [25, 26]. Beyond the structural support, EVs carry bioactive lipid mediators and enzymes involved in lipid metabolism [27, 28]. Dysregulated lipid metabolism has been strongly associated with ovarian cancer onset, progression, and therapeutic resistance [29, 30]. A deeper understanding of these molecular alterations in lipid metabolism can ultimately improve clinical outcomes of ovarian cancer patients by identifying diagnostics and prognostic biomarkers.

Recent studies have demonstrated that the lipid composition of EV membranes differs significantly from that of their parental cancer cells, reflecting distinct biological roles and their potential as biomarkers. For instance, EVs released from metastatic PC‐3 prostate cancer cells exhibit enrichment of cholesterols, long chain sphingomyelins (SMs), phosphatidylserine (PS), hexosylceramide (Hex3Cer), and lactosylceramide (LacCer) compared to their parent cells, indicating selective lipid sorting into vesicles [31]. Interestingly, PS‐enriched exosomes have been detected at significantly higher levels in blood of ovarian cancer patients compared to controls, aligning with the known overexpression of PS on tumor cell membrane and their release as plasma exosomes, suggesting PS as a minimally invasive diagnostic marker [32, 33]. Similarly, EVs from highly metastatic triple‐negative breast cancer (TNBC) cell lines were enriched with unsaturated diacylglycerol (DAG 18 : 1/20 : 2) relative to those from low metastatic breast cancer cells [34]. Lipidomic analysis in colorectal carcinoma EVs has shown elevated levels of sphingolipids, glycerolipids, sterol lipids, and glycerophospholipids such as plasmalogens and alkyl ethers [35]. In ovarian cancer, EVs derived from the SKOV‐3 cell line are found to be enriched in cholesterol esters, gangliosides, zymosterols, acylcarnitines, and specific lyso‐phospholipids (LPS, LPG, LPC, and LPI), while ceramides (Cer), phospholipids (PS, PI, PG, PE), SM, and diacylglycerols (DAG) were down‐regulated [36]. However, there is limited understanding of how lipids are sorted into EVs. Evidence suggests that sorting mechanisms for proteins and lipids into EVs operate independently, and the lipid sorting is more closely related to EV size and quantity [37]. These findings suggest the complexity and heterogeneity of lipid profiles in EVs across cancer cell types, supporting the importance of lipidomic profiling in understanding tumor biology and identifying disease‐specific biomarkers.

In this study, we performed comprehensive lipidomic profiling of EVs isolated from clinically relevant biofluids, including plasma and ascites from patients with high‐grade serous ovarian cancer, as well as from ovarian cancer cell lines, to identify EV‐associated lipid signatures accessible through minimally invasive sampling. An EV lipid panel was then selected and evaluated for biomarker performance in two independent plasma cohorts. Importantly, the cell lines used in this study represent key features of tumor heterogeneity, chemoresistance, and TP53 mutation status. Together, this integrated approach aimed to identify clinically informative EV lipid signatures that reflect tumor metabolism and heterogeneity, supporting their potential as biomarkers and therapeutic targets in ovarian cancer.

## 2 Materials and methods

### 2.1 Quality management system

All experimental procedures were conducted within an ISO17025 accredited (National Association of Testing Authorities, Australia) research facility. All data were recorded within a 21 Code of Federal Regulation part 11 compliant electronic laboratory notebook (Lab Archives, Carlsbad, CA 92008, USA).

### 2.2 Study participants

Plasma samples were obtained from 146 participants, comprising patients with benign adnexal masses (*n* = 50), healthy control women without evidence of malignant or benign gynecological disease (*n* = 50), and patients with ovarian cancer (*n* = 46). Ascites samples were obtained from patients with ovarian cancer (*n* = 7). This study was performed in accordance with the Declaration of Helsinki. Samples were collected between January 2022 and April 2025 and the study was approved by the Ethics committee of the University of Queensland and Mater Hospital (2023/HE002226), Human Research Ethics Committees at The University of Queensland (2005000785), and Royal Brisbane and Women's Hospital (RBWH 2005/022). The plasma samples and clinical data were obtained from the women's cancer Biobank, Mater Hospital, Brisbane, Australia. The experiments were conducted with the understanding and written informed consent of each patient. Whole blood samples were collected by venipuncture using EDTA tubes, and plasma was obtained by 10 min centrifugation at 2500 rpm. The obtained plasma was stored at −80 °C until further analysis. Ascites/peritoneal fluid samples were centrifuged at 300 ***g*** for 5 min at 4 °C to remove the cellular debris and any remaining tissue fragments. The supernatant was carefully collected, avoiding the cell pellet and any possible contamination. The precleared ascites samples were then aliquoted and stored at −80 °C until further experimental use.

### 2.3 Cell lines

The following ten cell lines were used as ovarian cancer models: SKOV‐3 (RRID:CVCL_0532), TOV112D (RRID:CVCL_3612), OVCAR‐3 (RRID:CVCL_0465), HeyA8 (RRID:CVCL_8878), OVCA‐420 (RRID:CVCL_3935), PEO1 (RRID:CVCL_2686), OV‐90 (RRID:CVCL_3768), PEO4 (RRID:CVCL_2690), OVCA‐429 (RRID:CVCL_3936), CAOV‐3 (RRID:CVCL_0201) along with control cell lines: Met5A (RRID:CVCL_3749), HEK293T (RRID:CVCL_0063), HOSE 17.1 (RRID:CVCL_7672). All cell lines were obtained from the American Type Culture Collection (ATCC; Manassas, VA, USA) and cultured according to the supplier's recommendations. Specifically, OVCAR‐3, SKOV‐3, HeyA8, CAOV‐3, PEO4, OVCA‐420 were grown in RPMI media (Cat No. 11835‐030; Gibco, Thermo Fisher Scientific, Waltham, MA, USA) with 10% fetal bovine serum (FBS) and 1% Antibiotic‐Antimycotic solution. The OVCA‐429, PEO1, HEK293T cell lines were grown and maintained in DMEM media (Cat No. 11995‐065; Gibco) with 10% FBS and Met5A, HOSE17.1, OV‐90 were grown in a 1 : 1 mixture of MCDB (Cat No. M6395; Sigma Aldrich, St. Louis, MO, USA) and Medium 199 (Cat No. 11150‐059; Gibco) with 1% Antibiotic‐Antimycotic solution. All cultures were maintained in a tri‐gas incubator at 37 °C with 5% CO_2_ and 8% O_2_ to mimic physiological oxygen levels. Prior to experimental use, each cell line was authenticated by short tandem repeat (STR) profiling. All experiments were performed with mycoplasma‐free cells. OVCAR‐3 and HOSE 17.1 cell lines used in this study were kindly provided by Prof. John Hooper's lab, Mater Hospital, The University of Queensland, Australia.

### 2.4 Plasma‐derived EV isolation

EVs were isolated from plasma samples using two pre‐clearing steps followed by size‐exclusion chromatography (SEC). A 120 μL of plasma sample was centrifuged at 1500 ***g*** for 10 min at 4 °C and supernatant was collected. Further, the obtained supernatant was centrifuged at 10 000 ***g*** for 10 min at 4 °C to remove large EVs. A 100 μL of plasma sample was used to isolate the EVs, and 10 μL crude plasma was kept for lipid extraction directly. A 100 μL of plasma sample was diluted 5 times with phosphate saline buffer (PBS), and SEC was performed for isolating the EVs using qEV Gen2 70 nm pore size columns (Izon Science Ltd.) using an automated fraction collector. The column was flushed with two columns of PBS before loading the supernatant obtained after centrifugation of 10 000 ***g***. Consistent with the manufacturer's specifications for the qEVoriginal Gen 2 70 nm column, the first 2.9 mL of void volume was discarded, and the subsequent three fractions of 400 μL each were collected as the EV‐containing fractions. Prior to fraction selection, immunoblot analysis of classical EV markers confirmed that these first three fractions were enriched for EVs. After each sample, columns were washed with 17 mL PBS after each 8.5 mL 0.5 m NaOH cleaning and stored in 0.05% ProClin200 storage solution. In a similar manner, EVs from ascites samples (500 μL) from each patient were isolated, concentrated down to 100 μL of EV‐enriched samples, and further characterized.

### 2.5 Preparation of cell‐derived EV samples

To prepare cells for EV isolation, ovarian cancer and control cell lines were cultured until they reached ~ 70% confluency. At this stage, cells were washed twice with PBS and then incubated in their respective culture media supplemented with 1% Antibiotic‐Antimycotic solution but without FBS for 48 h. After the incubation period, conditioned media were collected, and cell pellets from each cell line were retained for direct lipid extraction. EVs were isolated from conditioned media of ovarian cancer and control cell lines using preclearing process followed by SEC. The collected conditioned media was first centrifuged at 300 ***g*** for 10 min at 4 °C to remove cellular debris. The obtained supernatant was then centrifugated at 2000 ***g*** followed by 12 000 ***g*** for 10 min at 4 °C. The final supernatant was concentrated to 1 mL of conditioned media using 100 kDa molecular weight cut off (MWCO) Amicon Ultra centrifugal filters (Millipore, Burlington, MA, USA) and stored at −80 °C until further processing. SEC was performed as described in Section 2.4 to isolate EVs using 500 μL of conditioned medium collected from each cell line utilizing qEV Gen2 70 nm pore size columns (Izon Science Ltd., Christchurch, New Zealand). The obtained EV fractions were concentrated down to 100 μL using 100 kDa molecular weight cut off (MWCO) Amicon Ultra centrifugal filters (Millipore). The pooled EV samples from each triplicate cell lines were used for immunoblotting and cryo‐transmission electron microscopy (Cryo‐TEM).

### 2.6 Nanoparticle tracking analysis (NTA) of EVs

The concentration and size distribution of EVs from each ovarian cancer cell line (biological triplicates) and from ascites and plasma were analyzed by NTA using NanoSight NS500 (NTA 3.4‐Sample Assistant Build 3.4.4‐SA) (Nanosight Ltd, Malvern Instrument Ltd, Malvern, Worcestershire, UK) equipped with a highly sensitive scientific CMOS camera having 405 nm blue laser. Samples were diluted in the ratio of 1 : 200 in particle free PBS to have concentration within the instrument's recommended range (approximately 50 particles per frame) before loading. Prior to injection into the autosampler chamber, samples were vortexed and measurements were performed at 25 °C with a viscosity of 0. 89 cP. The camera level was set to 13 and five videos of 60s each were recorded per sample. The particle size (mean and mode) and concentration (particles/mL) were calculated from the videos using nta 3.4 software. Size distribution profiles were then plotted using graphpad prism version 9.4.1(681).

### 2.7 Protein estimation and immunoblotting analyses

The protein concentration of EVs and of cells, ascites, and plasma samples was estimated using the Pierce BCA Assay kit (Thermo Fisher) according to the manufacturer's protocol. The samples were diluted in 1 : 1 ratio in RIPA buffer and measured in triplicates against a serial BSA standard curve. Absorbance was read at 562 nm using SPECTROstar Nano microplate reader (BMG Labtech, Ortenberg, Germany) and protein concentrations (μg·mL^−1^) were interpolated from the standard curve. Equal amounts of protein were loaded for EVs, corresponding cell lysates and ascites, plasma samples. Protein samples were mixed with 10X Bolt sample reducing agent and 4X Bolt LDS sample buffer, then heated at 70 °C for 10 min. Protein bands were resolved on Bolt 4–12% Bis‐Tris‐Plus gel on SDS‐PAGE at 200 V for 30 min followed by wet transfer to Immobilon‐FL PVDF membrane (Millipore; 0.45 μm pore) at 100 V for 1 h. Membranes were blocked with Intercept blocking buffer (LI‐COR Biosciences, Lincoln, NE, USA) for 1 h at room temperature. Primary antibodies were diluted in Intercept antibody dilution buffer (LI‐COR Biosciences) and incubated overnight at 4 °C.The following antibodies were used: rabbit mAb to CD9 (13174S; 1 : 1000), rabbit mAb to CD81 (56039S; 1 : 1000), rabbit mAb to flotillin‐1 (18634S; 1 : 1000), rabbit mAb to Grp94 (D6X2Q) XP (20 292; 1 : 1000); all were purchased from cell Signaling Technology, Inc. The rabbit monoclonal anti‐ALIX (ab186728; 1 : 1000), rabbit monoclonal antibody to TSG101 (ab125011; 1 : 1000), rabbit recombinant monoclonal antibody to ApoA1 (ab52945; 1 : 1000) and bovine serum Albumin antibody (cat. No. ab192603; 1:1000 dilution; Abcam, Cambridge, UK), were purchased from Abcam. After three 5 min washes in 1× TBS with 0.1% Tween‐20 (TBS‐T), membranes were incubated with IRDye800‐conjugated goat anti‐rabbit IgG secondary antibody (LI‐COR Biosciences; 1 : 10000) for 1 h at room temperature. Blots were washed three times (10 min each) in TBS‐T washing buffer and imaged using ChemiDoc system with 700‐ and 800‐nm channels.

### 2.8 Cryogenic transmission electron microscopy (cryo‐TEM)

EV samples were examined by cryo‐TEM at the Centre for Microscopy and Microanalysis, The University of Queensland. The pooled EV preparations were loaded onto carbon‐coated perforated formvar film supported on a 200‐mesh copper TEM grid using a Leica EM GP2 robotic vitrification system, operated at 22 °C and 95% relative humidity. Further, 3 μL of EV samples  was applied to the grid, excess liquid was blotted for 3–3.5 s and the grid was rapidly plunged into liquid ethane cooled to approximately 182.8 °C to achieve vitreous ice. Grids were stored in liquid nitrogen until imaging. Further, imaging was performed on a Jeol Cryo ARM200 (JEM‐Z200FSC) transmission electron microscopy equipped with a cold field emission gun and an in‐column omega energy filter. The microscope was operated at 200 kV, and images were recorded under low‐dose conditions in the zero‐loss energy mode with a 20 eV energy filter setting. The frozen‐hydrated samples were maintained at −176 °C during data collection. Images were acquired using a Gatan K2 direct electron detector and serialem automated acquisition software.

### 2.9 Lipid extraction‐solid phase extraction (SPE)

Lipid extraction from cell lines, biofluids and respective EVs was performed using Bond Elut Lipid Extraction SPE 2 mL 96‐well plates (cat No. 5610‐2043; Agilent, Alabaster, AL, USA) following the manufacturer's recommended protocol. A 100 μL aliquot of each sample (cell lysate, crude ascites, plasma and concentrated EVs) was spiked in with 5 μL of 2×‐diluted UltimateSPLASH™ ONE Mass Spec Standard (cat No. 330820; Avanti POLAR LIPIDS, Alabaster, AL, USA). All solvents of LC‐MS grade were used in lipid extraction. Protein precipitation was achieved by adding 900 μL of ice‐cold ACN/MeoH (99 : 1, v/v), vortexing for 30s, and sonicating on ice for 10 min. The entire homogenate (including protein pellet) was transferred directly to the SPE plate without centrifugation and loaded under gravity with a flow rate of ~ 3–5 drops per second to ensure efficient lipid retention on the EMR‐Lipid sorbent. The plate was washed twice with 1 mL of ACN/water (9 : 1, v/v), before loading and flow‐through was discarded. After washing, the plate was dried under high vacuum pressure and the waste reservoir was removed. A 2 mL glass‐coated collection plate was placed below and the lipids were eluted twice with 1 mL of chloroform/MeOH (1 : 1, v/v). The eluted lipids were dried under a gentle stream of N2 at room temperature, then reconstituted in 100 μL of 100% methanol by vortexing for 2 min and sonicating for 10 min at room temperature. The reconstituted samples were centrifuged at 2000× ***g*** for 5 min, and the clear supernatant was transferred into 2 mL vials with glass inserts. A 50 μL aliquot per sample was sent to the LC–MS facility at the UQ Frazer Institute, Brisbane, Australia for lipidomic analysis.

### 2.10 LC–MS/MS‐ based lipid class analysis and MS‐DIAL data processing

Lipidomic data were generated in two analytical phases. In the initial phase, lipid extracts were analyzed by direct infusion using a SCIEX 5600+ qTOF mass spectrometer with information‐dependent acquisition (IDA) MS/MS. The data were processed in LipidView (SCIEX) to obtain an overview of the major lipid classes and subsequently analyzed using the LipidSuite web server. In the expanded phase for biomarker panel, all samples were analyzed on an Agilent 1290 Infinity UHPLC system connected to a SCIEX TripleTOF 5600 mass spectrometer fitted with a DuoSpray ion source and operated in positive‐ and negative‐ion electrospray modes. Data were acquired and the instrument was controlled using analyst tf software version 1.8.1. Separation was achieved on a Kinetex C18 column (2.6 μm, 100 Å, 50 × 21 mm; Phenomenex, Torrance, CA, USA) at 50 °C, using a mobile phase of (A) 60% acetonitrile in water with 10 mm ammonium acetate and (B) 90% isopropanol in acetonitrile with 10 mm ammonium acetate. The flow rate was set to 0.45 mL·min^−1^, with the gradient run from 35% B to 98% B over 7.2 min and then re‐equilibrated at 35% B for 7.3 min and then maintained until 8.8 min. Injection volumes were 3 μL for biofluid (ascites and plasma lipid samples) and cell‐derived lipid samples and 5 μL for the corresponding EV lipid samples.

The mass spectrometer was operated with GS1; 40 psi, GS2; 40 psi, CUR; 30 psi, source temperature 450 °C, ion spray voltage floating of +5500 V (positive) and −4500 (negative), and delustering potentials of +100 V and −100 V, respectively. Mass calibration was performed every 24 injections using X500 ESI calibration solutions (5 μL). Data was acquired using SWATH data‐independent acquisition (DIA) with MS scheduled from 1.0 to 7.0 min. In positive‐ion mode, TOF‐MS scans covered m/z 400–1100 (150 ms accumulation), with ~ 30 Da precursor isolation windows and a cycle time of 1.07 s (34.9 eV collision energy, 20 eV spread). In negative‐ion mode, scans covered m/z 350–1000 (150 ms), with ~ 30 Da windows and a cycle time of 0.98 s (−29.5 eV collision energy, 30 eV spread). High‐sensitivity TOF‐MS and MS/MS spectra were acquired for lipid identification.

For quality control, analytical reproducibility was assessed using pooled biological QC samples prepared for each batch and injected at the beginning, middle, and end of each batch. A blank 100% methanol was injected after every five samples to monitor carryover. Instrument performance and mass accuracy were continuously monitored throughout the analytical sequence by routine calibration and QC sample evaluation.

Lipidomic data were processed using ms‐dial (version 5.5.260323). Raw SWATH data acquired on the SCIEX TripleTOF 5600 were imported as centroid LC/MS chromatographic data collected in positive‐ and negative‐ ion modes using CID/HCD fragmentation, and the target omics setting was selected for lipidomics. Mass accuracy parameters were set to 0.01 Da for MS1 and 0.025 Da for MS2. Peak detection was performed at a minimum peak height of 1000 amplitude, and a mass slice width of 0.1 Da. Deconvolution was conducted using a sigma window value of 0.5, with MS/MS abundance and relative abundance cutoffs set to 0 amplitude and 0%, respectively. Alignment was performed using the mid‐QC samples as the reference file, with a retention time tolerance of 0.1 min and an MS1 tolerance of 0.015 Da. Normalization was performed in MS‐DIAL using total ion current (TIC) for each sample.

### 2.11 Statistical analysis

Lipid species abundance was quantified based on extracted peak areas from MS‐DIAL output after TIC normalization. Unknown features and lipid species with low identification scores were removed prior to statistical analysis to ensure high‐confidence annotations. The filtered lipidomic dataset was then log_2_‐transformed as log_2_ (*x* + 1) to approximate a normal distribution, and all downstream analyses were performed in r. Biomarker‐based classification and performance evaluation were performed by leave‐one‐out cross validation in python using pycaret 3.3.2.

## 3 Results

### 3.1 Isolation and characterization of EVs from biofluids of HGSOC patients and ovarian cancer cell lines

Clinical characteristics of patients with HGSOC providing plasma and ascites samples are summarized in Table 1.

**Table 1:** Clinical characteristics of the patients involved in this study.

Characteristic	Overall *N* = 73^a^	Control *N* = 25^a^	Benign *N* = 25^a^	Cancer *N* = 23^a^
**(A) Training: Plasma cohort**				
Age at diagnosis (Years)	51 (40, 61)	41 (34, 48)	55 (44, 68)	54 (45, 70)
Age at sample collection (Years)	52 (40, 61)	41 (34, 48)	55 (44, 68)	55 (45, 70)
CA125 (U·mL^−1^)	8 (5, 39)	7 (5, 11)	6 (4, 18)	314 (50, 1782)
Tumor stage				
I	2 (8.7%)	0 (NA%)	0 (NA%)	2 (8.7%)
II	0 (0%)	0 (NA%)	0 (NA%)	0 (0%)
III	11 (48%)	0 (NA%)	0 (NA%)	11 (48%)
IV	5 (22%)	0 (NA%)	0 (NA%)	5 (22%)
Unknown	5 (22%)	0 (NA%)	0 (NA%)	5 (22%)
Histology type				
Other	0 (0%)	0 (NA%)	0 (NA%)	0 (0%)
Serous	23 (100%)	0 (NA%)	0 (NA%)	23 (100%)
Neoadjuvant chemotherapy				
N	15 (83%)	0 (NA%)	0 (NA%)	15 (83%)
Y	3 (17%)	0 (NA%)	0 (NA%)	3 (17%)
BRCA status				
BRCA mutation detected	5 (6.8%)	0 (0%)	0 (0%)	5 (22%)
No mutations detected	9 (12%)	0 (0%)	0 (0%)	9 (39%)
Not tested	4 (5.5%)	0 (0%)	0 (0%)	4 (17%)
Unknown/Not tested	55 (75%)	25 (100%)	25 (100%)	5 (22%)
**Training: Ascites cohort**				
**Characteristic**				**Ascites *N* = 7** ^a^
Age at diagnosis (Years)	0 (NA%)	0 (NA%)	0 (NA%)	61 (44, 74)
CA125 (U·mL^−1^)	0 (NA%)	0 (NA%)	0 (NA%)	1199 (672, 3720)
Tumor stage				
I	0 (NA%)	0 (NA%)	0 (NA%)	0 (0%)
II	0 (NA%)	0 (NA%)	0 (NA%)	0 (0%)
III	0 (NA%)	0 (NA%)	0 (NA%)	0 (0%)
IV	0 (NA%)	0 (NA%)	0 (NA%)	4 (57%)
Unknown	0 (NA%)	0 (NA%)	0 (NA%)	3 (43%)
Histology type				
Other	0 (NA%)	0 (NA%)	0 (NA%)	0 (NA%)
Serous	0 (NA%)	0 (NA%)	0 (NA%)	7 (100%)
Neoadjuvant chemotherapy				
N	0 (NA%)	0 (NA%)	0 (NA%)	2 (29%)
Y	0 (NA%)	0 (NA%)	0 (NA%)	3 (42%)
Unknown	0 (NA%)	0 (NA%)	0 (NA%)	2 (29%)
BRCA status				
BRCA mutation detected	0 (NA%)	0 (NA%)	0 (0%)	0 (0%)
No mutations detected	0 (NA%)	0 (NA%)	0 (0%)	4 (57%)
Not tested	0 (NA%)	0 (NA%)	0 (0%)	2 (29%)
Unknown/not tested	0 (NA%)	0 (NA%)	0 (0%)	1 (14%)
**(B) Verification: Plasma cohort**				
Age at diagnosis (Years)	54 (42, 65)	38 (27, 48)	58 (48, 69)	64 (53, 74)
Age at sample collection (Years)	54 (42, 65)	38 (27, 48)	58 (48, 69)	64 (53, 74)
CA125 (U·mL^−1^)	7 (4, 14)	6 (5, 8)	5 (3, 8)	328 (34, 1154)
Tumor stage				
I	0 (0%)	0 (NA%)	0 (NA%)	0 (0%)
II	2 (8.7%)	0 (NA%)	0 (NA%)	2 (8.7%)
III	17 (74%)	0 (NA%)	0 (NA%)	17 (74%)
IV	1 (4.3%)	0 (NA%)	0 (NA%)	1 (4.3%)
Unknown	3 (13%)	0 (NA%)	0 (NA%)	3 (13%)
Histology type				
Other	1 (4.3%)	0 (NA%)	0 (NA%)	1 (4.3%)
Serous	22 (96%)	0 (NA%)	0 (NA%)	22 (96%)
Neoadjuvant chemotherapy				
N	18 (90%)	0 (NA%)	0 (NA%)	18 (90%)
Y	2 (10%)	0 (NA%)	0 (NA%)	2 (10%)
BRCA status				
BRCA mutation detected	3 (4.1%)	0 (0%)	0 (0%)	3 (13%)
No mutations detected	9 (12%)	0 (0%)	0 (0%)	9 (39%)
Not tested	8 (11%)	0 (0%)	0 (0%)	8 (35%)
Unknown/not tested	53 (73%)	25 (100%)	25 (100%)	3 (13%)

^a^ Median (Q1, Q3); *n* (%).

To obtain a comprehensive and representative lipidomic profile of ovarian cancer‐associated EVs, vesicles were isolated and characterized from ascites (*n* = 7) and plasma samples of HGSOC patients (*n* = 46) with control (*n* = 50) and benign (*n* = 50) along with 13 cell lines, including ovarian cancer cells SKOV‐3, TOV112D, OVCAR‐3, HeyA8, OVCA‐420, PEO1, OV‐90, PEO4, OVCA‐429, CAOV‐3, as well as non‐cancer cell lines, immortalized human ovarian surface epithelium cell line HOSE 17.1, immortalized human mesothelial cell line Met5A, and immortalized human embryonic kidney 293T cell line, as described in Material and methods and shown in Fig. 1A. The characteristics of these ovarian cancer and control cell lines are summarized in Table S1. Prior to characterization and selection of EV fractions in this study cohort, immunoblot analysis of classical EV markers demonstrated that the first three SEC fractions isolated from control plasma samples were enriched for EVs relative to subsequent fractions (4–13), and these fractions were therefore considered as the EV‐containing fractions and used for downstream analyses (Fig. S1). Plasma‐derived EVs from control, benign, and HGSOC patients showed similar size distribution, with mode particle sizes of 91.77 ± 1.21, 95.86 ± 1.35, and 97.34 ± 1.52 nm, respectively (Fig. 1B). Individual particle concentrations and mode sizes for each patient's plasma‐derived EVs are shown in Fig. S2. Further, immunoblotting confirmed the enrichment of EV markers, including transmembrane proteins (Category I: CD9, CD81) and cytosolic proteins (Category II: Flotillin‐1, Alix), while non‐EV markers (Category III: ApoA1 and Albumin) were used as contaminant markers (Fig. 1C). Cryo‐TEM further confirmed the vesicular morphology of plasma EVs, revealing intact spherical structures with lipid bilayers (Fig. 1D).

**Fig. 1:**
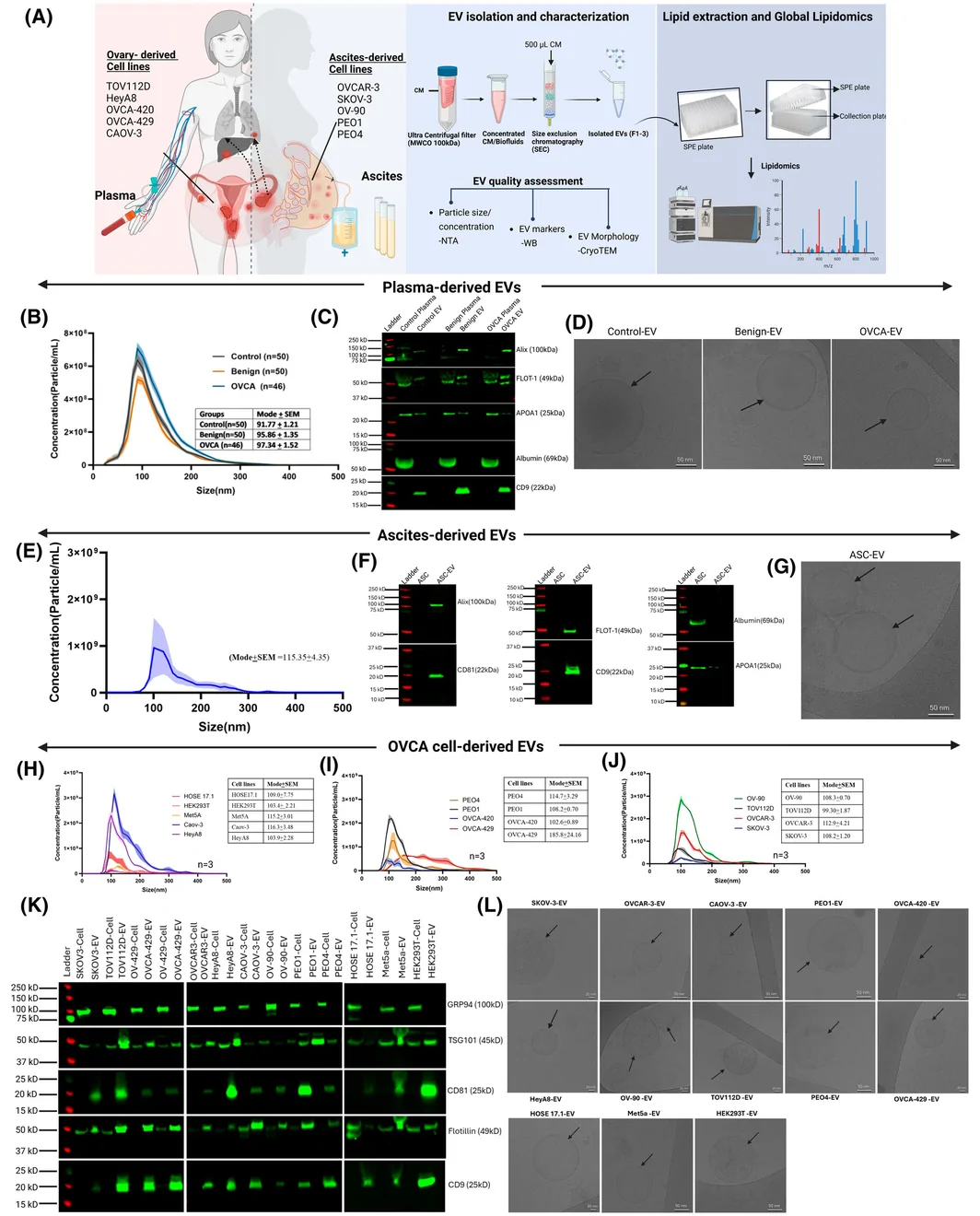
Isolation and characterization of extracellular vesicles (EVs). (A) Workflow summarizing EV isolation and characterization from ovarian cancer cell lines, non‐cancer control cell lines and ascites/plasma of high‐grade serous ovarian cancer (HGSOC) patients, followed by solid‐phase lipid extraction for untargeted lipidomic analysis. Characterization of biofluid‐derived and ovarian cancer cell‐line‐derived EVs. Created in BioRender.com/z6cab3w. (B, E, H–J) Nanoparticle tracking analysis (NTA) showing particle size distribution of isolated EVs from control (*n* = 50), benign (*n* = 50) and OVCA (*n* = 46), patient's plasma and ascites of HGSOC patients (*n* = 7), ovarian cancer (*n* = 10) and non‐cancer cell lines (*n* = 3). Data presented as mode size± SEM. The pooled EV preparations were utilized for immunoblot and cryo‐EM analysis (C, F & K) Immunoblot analysis of EV protein markers, confirming the presence of canonical transmembrane proteins (CD81, CD9; category I) and cytosolic proteins (Flotillin‐1, TSG101, Alix; category II) and absence of non‐EV contaminants (GRP94; category IV, albumin and ApoA1; category III). Cryo‐EM images of (D) plasma‐derived EVs (control, benign, OVCA; scale bar 50 nm), (G) ascites‐derived EVs (scale bar 50 nm) and (L) Ovarian cancer and non‐cancer cell line derived EVs displaying intact lipid bilayer structures. The scale bars: 50 nm (OVCAR‐3, CAOV‐3, PEO1, PEO4, HEK293T, Met5A); 20 nm (SKOV‐3, OVCA‐420, HeyA8, OV‐90, TOV112D, OVCA‐429, HOSE 17.1).

Similarly, ascites‐derived EVs exhibited a mode particle size of 115.35 ± 4.35 nm (Fig. 1E). The presence of the EV markers Alix, CD81, CD9, and Flotillin confirmed EV enrichment (Fig. 1F), while cryo‐TEM demonstrated characteristic spherical, bi‐layered morphology of EVs (Fig. 1G).

EVs derived from ovarian cancer and control cell lines displayed mode particle sizes ranging from approximately 99.30 to 185.8 nm (Fig. 1H–J). Individual particle concentrations and mode sizes for each cell‐line‐derived EVs are shown in Fig. S2. In these EVs, expressions of classical EV markers, including TSG101, CD81, CD9, and Flotillin‐1, confirmed EV enrichment, while ER resident protein GRP94 (Category IV) was assessed as a non‐EV marker for cell‐derived EV preparations (Fig. 1K). Cryo‐TEM further verified the presence of spherical, bi‐layered vesicles, consistent with the NTA measurements (Fig. 1L).

The intensities of EV‐associated markers varied depending on the cellular source and sample origin. According to Minimum information for studies of extracellular vesicles (MISEV) guidelines, these characterization markers are categorized as transmembrane (category I), cytosolic proteins (category II), and markers of non‐EV structures (category III and IV). In summary, these results confirm the effective enrichment and isolation of EVs from both conditioned media and ascitic fluid/plasma from HGSOC patients. Subsequently, solid phase extraction (SPE) based lipid extraction was done from cells, biofluid from ovarian cancer patients, and their corresponding EVs.

We next performed comprehensive lipidomic profiling followed by differential analysis comparing OVCA cells to their corresponding EVs, ascitic fluid to EVs, and plasma to EVs.

### 3.2 Lipidomic profiling reveals a source‐specific lipid signature enriched in ovarian cancer‐associated extracellular vesicles

To determine whether EVs carry a lipid cargo that is compositionally distinct from their cell or fluid of origin, we performed untargeted lipidomics on EVs isolated from OVCA cell lines, control cell lines, ascites, and plasma from women with ovarian cancer, and compared each EV lipidome to that of its source material (Tables S2– S5). Volcano plots of the four pairwise comparisons (Fig. 2A–D) identified widespread lipid redistribution between EVs and their source: 213 lipids were enriched and 184 depleted in OVCA‐cell‐derived EVs relative to OVCA cells (Fig. 2A); 210 lipids were enriched and 173 depleted in control‐cell‐derived EVs relative to control cells (Fig. 2B); 153 lipids were enriched and 609 depleted in ascites‐derived EVs relative to whole ascites (Fig. 2C); and 325 lipids were enriched and 323 depleted in EVs relative to matched OVCA plasma (Fig. 2D). Across all four comparisons, a larger proportion of lipids were depleted from, rather than enriched in, EVs relative to their source — an effect most pronounced for ascites, where roughly four times as many lipids were depleted as enriched.

**Fig. 2:**
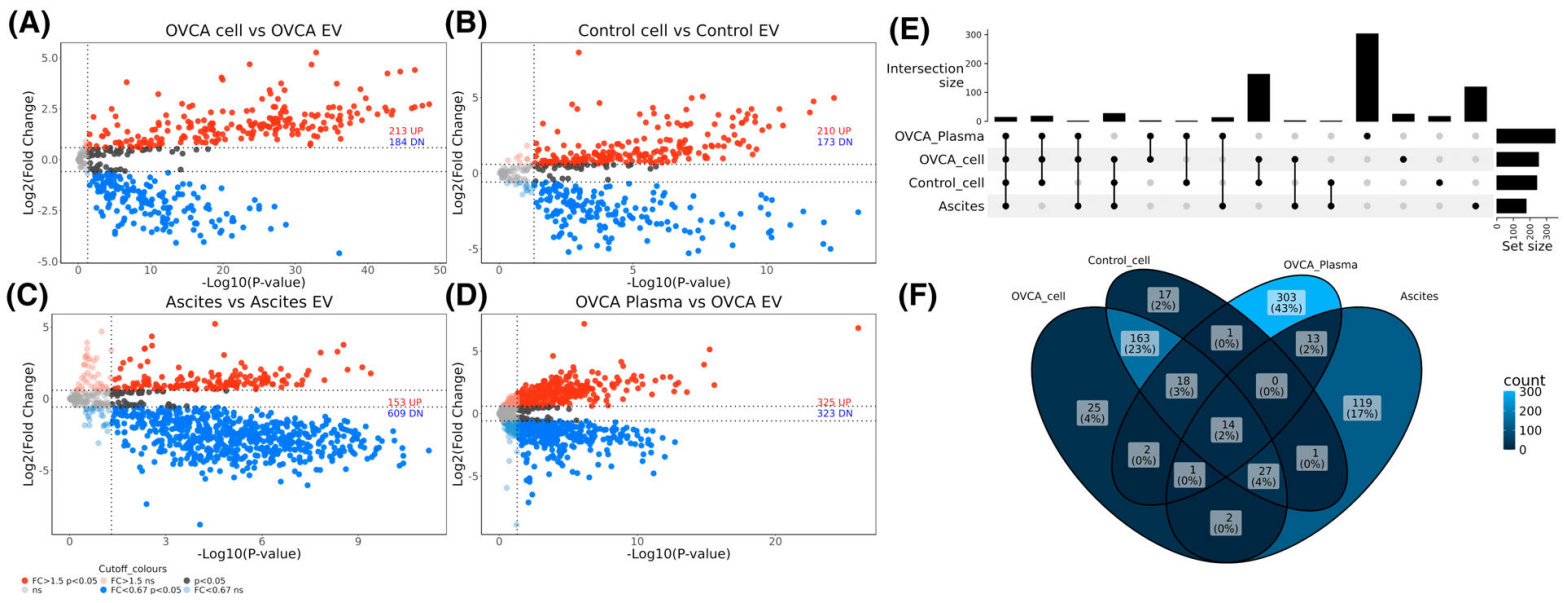
Lipidomic profiling identifies lipid species enriched in extracellular vesicles relative to their cellular and fluid sources. (A–D) Volcano plots comparing the lipidome of EVs to that of their source material: (A) OVCA cells vs. OVCA‐cell‐derived EVs, (B) control cells vs. control‐cell‐derived EVs, (C) whole ascites vs. ascites‐derived EVs, and (D) OVCA plasma vs. OVCA‐plasma‐derived EVs. For each comparison, log_2_(fold change) is plotted against −log_10_(*P*‐value); points are colored by significance and fold‐change cutoff (red, FC > 1.5 and *P* < 0.05; pink, FC > 1.5, not significant; dark gray, *P* < 0.05 only; light gray, not significant; blue, FC < 0.67 and *P* < 0.05; light blue, FC < 0.67, not significant). Positive values indicate lipids enriched in EVs relative to the source; negative values indicate lipids depleted from EVs (enriched in the source). The number of significantly up‐ and down‐regulated lipids is indicated in each panel. (E) UpSet plot showing the size and composition of intersections among the EV‐associated lipid sets from OVCA cells, control cells, ascites, and OVCA plasma; set sizes are shown in the adjoining bar chart. (F) Four‐way Venn diagram of the same lipid sets, with the number and percentage of lipids unique to or shared across each combination of sample types.

We next asked whether the same EV‐associated lipids were shared across sample types or specific to a given source. An UpSet plot (Fig. 2E) and complementary four‐set Venn diagram (Fig. 2F) of the EV‐associated lipid sets from OVCA cells, control cells, ascites, and OVCA plasma showed that only 14 lipids (2%) were common to all four conditions, indicating that most EV lipid signatures are source‐specific rather than universal. The largest shared subset was between OVCA‐cell‐ and control‐cell‐derived EVs (163 lipids, 23%), suggesting substantial overlap in the core EV lipidome of cultured cells regardless of malignant status. In contrast, plasma‐ and ascites‐derived EVs each contributed a large proportion of lipids not seen in the other three sets (303 lipids, 43% unique to plasma; 119 lipids, 17% unique to ascites), consistent with the more complex biofluid matrix from which these EVs were isolated. Smaller three‐ and four‐way intersections (≤ 27 lipids each) further indicate limited concordance between cell‐line‐derived and biofluid‐derived EV lipidomes.

The overlap between OVCA‐cell‐derived EVs and OVCA‐plasma‐derived EVs was limited: only two shared lipid species were identified, DG 36 : 2 (DG 18 : 1_18 : 1) and SM 39 : 1; O2 (SM 18 : 1; O2/21 : 0), suggesting these as candidate lipid markers detectable both in tumor cells and in the circulation (Fig. 2F). Similarly, only two lipid species — DG 34 : 1 and VAE 20 : 1—were shared between OVCA‐cell‐derived EVs and ascites‐derived EVs. Notably, OVCA‐plasma‐derived and ascites‐derived EVs shared 13 lipid species, the largest pairwise overlap between the two biofluid‐derived EV sets, including one ceramide (Cer 51 : 7;O2/Cer 15 : 2;O2/36 : 5), two diacylglycerol (DG 19 : 0, DG 28 : 0 | DG 14 : 0_14 : 0), one ether‐phosphatidylcholine (PC O‐35 : 6/PC O‐16 : 2_19 : 4), one sphingomyelin (SM 43 : 8;O3), five triglycerides (TG 38 : 0/TG 10 : 0_12 : 0_16 : 0; TG 40 : 1/TG 8 : 0_14 : 0_18 : 1; TG 44 : 0/TG 13 : 0_15 : 0_16 : 0; TG 45 : 3/TG 14 : 1_15 : 1_16 : 1; TG 46 : 2/TG 14 : 0_16 : 0_16 : 2), and three vinyl ether lipids (VAE 17 : 3, VAE 18 : 3, VAE 19 : 3) (Fig. 2F). This substantial overlap suggests that a considerable proportion of circulating lipids are also present within the ascitic microenvironment.

OVCA‐plasma‐derived EVs showed significantly higher levels of specific phosphatidylcholines (PC 34 : 1; PC 33 : 3/PC 16 : 0_17 : 3; PC 34 : 4/PC 14 : 0_20 : 4) and sphingomyelin (SM 36 : 1;O2/SM 18 : 1;O2/18 : 0), along with several triglycerides (TG 38 : 1/TG 8 : 0_12 : 0_18 : 1; TG 50 : 1/TG 16 : 0_18 : 0_16 : 1; TG 52 : 4/TG 16 : 0_18 : 1_18 : 3; TG 41 : 1/TG 9 : 0_15 : 0_17 : 1; TG 41 : 0/TG 8 : 0_16 : 0_17 : 0; TG 32 : 0/TG 5 : 0_5 : 0_22 : 0). In contrast, whole OVCA plasma was enriched in ceramides Cer 40 : 0;O3/Cer 14 : 0;O2/26 : 0 (2OH); Cer 47 : 4;O3; Cer 36 : 0;O3/Cer 13 : 0;O3/23 : 0; Cer 42 : 0;O3/Cer 16 : 0;O2/26 : 0 (2OH), diacylglycerols (DG 38 : 0, DG 46 : 8), and specific phosphatidylcholines and phosphatidylglycerols (PC 40 : 7, PC 41 : 0), reflecting selective lipid enrichment in EVs relative to the fluid from which they were isolated (Fig. 2D) (Table S4). Similarly, OVCA‐cell‐derived EVs exhibited higher abundances of ether‐linked triglycerides (TG O‐36 : 4/TG O‐7 : 0_11 : 0_18 : 4; TG O‐36 : 3/TG O‐18 : 3_9 : 0_9 : 0), triglycerides (TG 49 : 0 | TG 16 : 0_16 : 0_17 : 0, TG 45 : 2 | TG 15 : 0_14 : 1_16 : 1, TG 37 : 0 | TG 8 : 0_14 : 0_15 : 0) and diacylglycerols (DG 32 : 3, DG 30 : 0, DG 33 : 0) relative to their parental cells, whereas the OVCA cells themselves were enriched in phospholipids (PS 37 : 2; PC 40 : 5/PC 20 : 1_20 : 4; PC 38 : 2/PC 18 : 1_20 : 1; PC 35 : 2/PC 17 : 1_18 : 1; PC 34 : 2; PC 33 : 2; PC 32 : 1) and ceramides (Cer 42 : 1; O2/Cer 16 : 1; O2/26 : 0; Cer 43 : 1;O4/Cer 17 : 1; O3/26 : 0 (2OH); Cer 45 : 2;O3/Cer 18 : 2; O2/27 : 0) (Fig. 2A) (Table S2).

Ascitic fluid itself showed predominant enrichment of phosphatidylcholines (PC 42 : 10 | PC 20 : 4_22 : 6, PC 40 : 6 | PC 18 : 1_22 : 5, PC 40 : 7 | PC 18 : 1_22 : 6, PC 40 : 9 | PC 20 : 4_20 : 5, PC 38 : 5, PC 36 : 4) and ether‐phosphatidylcholines (PC O‐), whereas ascites‐derived EVs were enriched mainly in diacylglycerols (DG 30 : 3, DG 29 : 0, DG 35 : 3, DG 34 : 3, DG 31 : 3, DG 32 : 1), vinyl ether lipids (VAE 18 : 0, VAE 17 : 0, VAE 15 : 3), triglycerides (TG 51 : 0;O|TG 16 : 0_17 : 0_18 : 0;O), and sterol lipids (Fig. 2C) (Table S5). This pattern suggests selective packaging of lipid species into EVs, with ascitic fluid favoring phospholipid‐based compositions while ascites‐derived EVs contain relatively higher levels of neutral and storage lipids. Together, these analyses define a shortlist of lipid species that are consistently and specifically enriched in ovarian‐cancer‐associated EVs, which we next evaluated for biomarker potential.

### 3.3 An EV‐discovered lipid panel enhances CA125 diagnostic performance in EVs and whole plasma

To evaluate the diagnostic potential of the EV‐enriched lipid panel identified through discovery lipidomics, we assembled a clinical cohort of 146 women comprising healthy controls, women with benign adnexal masses, and women with ovarian cancer (Fig. 3, top). This cohort was divided into two independent, equally sized sets: a Selection/Training cohort (*n* = 73) used to identify the optimal EV‐derived lipid panel and its combination with the established biomarker CA125, and a Verification cohort (*n* = 73) used to confirm performance in an independent sample set. Each cohort comprised 50 non‐cancer samples (25 controls, 25 benign adnexal masses) and 23 ovarian cancer samples, allowing three pairwise diagnostic comparisons in each cohort: controls vs. ovarian cancer, benign adnexal masses vs. ovarian cancer, and combined controls‐and‐benign vs. ovarian cancer.

**Fig. 3:**
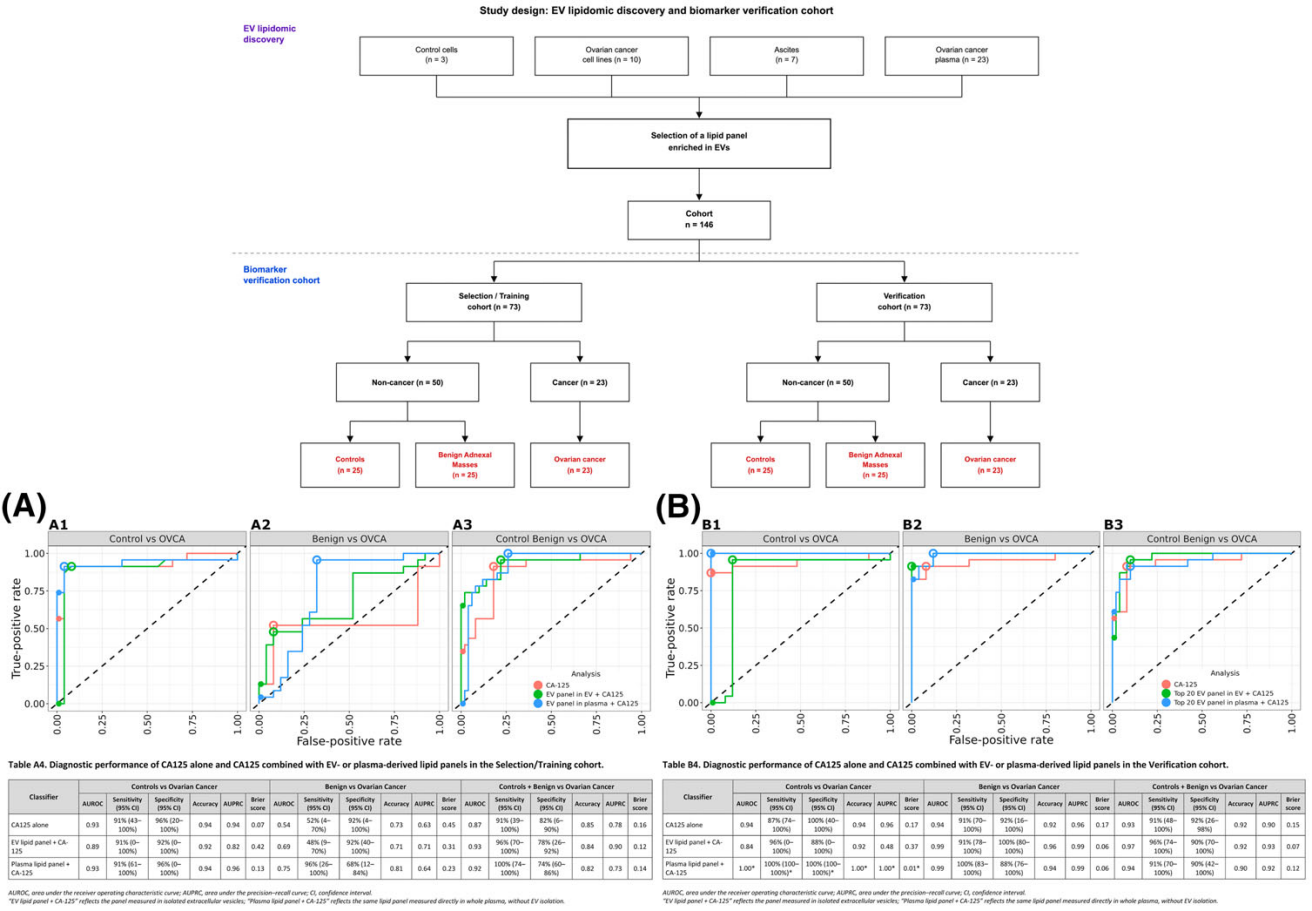
Study design and diagnostic performance of CA125 alone versus CA125 combined with EV‐ or plasma‐derived lipid panels. Top: EVs isolated from four sample types, control cells (*n* = 3), OVCA cell lines (*n* = 10), ascites (*n* = 7), and OVCA plasma (*n* = 23), were used to identify a lipid panel enriched in EVs relative to source material (Fig. 2). This panel was evaluated in a clinical cohort of 146 women, divided into a Selection/Training cohort (*n* = 73) and an independent Verification cohort (*n* = 73), each comprising 50 non‐cancer samples (25 controls, 25 benign adnexal masses) and 23 ovarian cancer samples (red text indicates final analytic groups). (A) Training and (B) Verification cohort ROC curves (A1–A3, B1–B3) and diagnostic performance metrics (A4, B4) for three classifiers, CA125 alone (red), EV lipid panel + CA125 (green), and plasma lipid panel + CA125 (blue), across three comparisons: controls vs. ovarian cancer, benign adnexal masses vs. ovarian cancer, and combined controls‐and‐benign vs. ovarian cancer. Dashed diagonal lines indicate chance‐level performance. A4 and B4 report AUROC, sensitivity and specificity (95% CI), accuracy, AUPRC, and Brier score for each classifier and comparison.

The lipid panel itself was selected entirely from the EV lipidome; we then asked whether this same panel retained diagnostic value when measured in two different ways: (1) in EVs isolated from patient plasma, replicating the discovery workflow, and (2) directly in whole plasma without an EV isolation step, testing whether the signal survives a simpler, more clinically practical workup. Three classifiers were therefore compared by ROC analysis in both cohorts: CA125 alone; the EV panel + CA125, measured in isolated EVs; and the same EV panel + CA125, measured directly in whole plasma (Fig. 3A,B).

In the Training cohort (Fig. 3A), CA125 alone discriminated controls from ovarian cancer well (AUROC 0.93) but performed poorly for benign adnexal masses vs. ovarian cancer (AUROC 0.54). Adding the EV panel to CA125, measured in isolated EVs, improved discrimination of benign vs. ovarian cancer (AUROC 0.69) and, more markedly, of the combined controls‐and‐benign vs. ovarian cancer comparison (AUROC 0.93 vs. 0.87 for CA125 alone). The same EV panel measured directly in whole plasma performed comparably to CA125 alone for controls vs. ovarian cancer (AUROC 0.93) and moderately improved benign vs. ovarian cancer discrimination (AUROC 0.75 vs. 0.54), indicating that the panel's diagnostic signal is at least partly retained even without EV isolation.

These improvements were more pronounced in the independent Verification cohort (Fig. 3B). CA125 alone performed consistently across all three comparisons (AUROC 0.93–0.94). Adding the EV panel measured in isolated EVs improved performance for benign vs. ovarian cancer (AUROC 0.99 vs. 0.94) and the combined comparison (AUROC 0.97 vs. 0.93), with lower Brier scores indicating better‐calibrated predictions; performance for controls vs. ovarian cancer with the EV‐isolated panel was comparatively lower (AUROC 0.84), with a wide confidence interval reflecting the small sample size in that comparison. The same panel measured directly in whole plasma also outperformed CA125 alone for benign vs. ovarian cancer (AUROC 0.99) and the combined comparison (AUROC 0.94); with reported AUROC of 1.00 for controls vs. ovarian cancer. Across both measurement approaches, CA125 remained among the top contributing features by SHAP importance, alongside a recurring set of ceramides, acylhexosylceramides, and bile‐acid‐conjugated lipid species; indicating the EV‐derived panel adds diagnostic information beyond CA125 regardless of whether EV isolation is performed.

Together, these results show that an EV‐discovered lipid panel, combined with CA125, improves discrimination of ovarian cancer from benign and non‐cancer samples beyond CA125 alone, whether the panel is measured in isolated EVs or directly in whole plasma, with the greatest benefit for distinguishing ovarian cancer from benign adnexal masses.

### 3.4 Storage and signaling lipids are selectively enriched in TP53 mutant ovarian cancer EVs

TP53 mutations are a common driver of high‐grade serous ovarian cancer, present in the majority of tumors (96% of cases) [38, 39]. We next examined the relationship between TP53 mutation status and lipid signaling. For this analysis, we selected TP53‐mutated cell lines (OVCAR‐3, OV‐90, PEO1, PEO4, TOV112D, CAOV‐3) along with TP53‐wildtype (WT) cell lines (OVCA‐429, OVCA420, HeyA8), with the workflow shown in Fig. 4A. Differential lipidomic analysis between TP53‐mutant and WT groups revealed distinct lipid signatures in both cells and their EVs (Fig. 4B,C) (Tables S6.1 and S6.2). In TP53 mutant‐EVs, specific enrichment of seven unique lipid species, such as TAG (58 : 12, 38 : 2), NAPE (34 : 3, 52 : 12), MADAG (42 : 1, 44 : 6), and LPS 30 : 0, was observed (Fig. 4D).

**Fig. 4:**
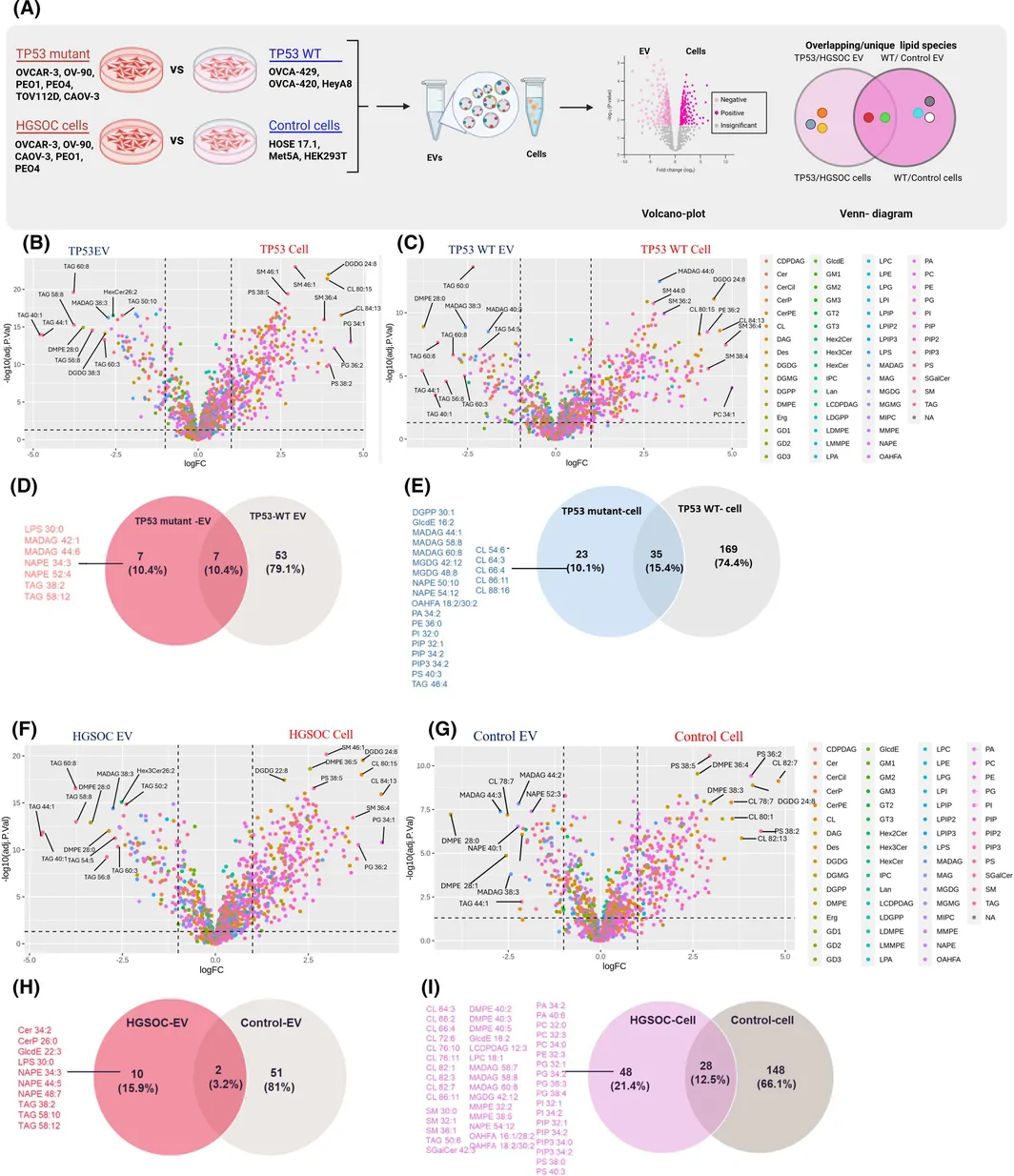
Lipidomic alterations associated with TP53 mutation and high‐grade serous ovarian phenotype. (A) Workflow showing differential lipid profiling using LipidSuite and overlapping analysis of EVs and cells from TP53 mutated/WT and high‐grade serous/control cell lines. Created in BioRender.com/spwb2xz. Volcano plot (B) comparing lipidomic profiles of EVs and cells from TP53 mutant ovarian cancer cell lines (OVCAR‐3, OV‐90, PEO1, PEO4, TOV112D, CAOV‐3) (C) comparing lipidomic profiles of WT TP53‐EVs and TP53‐WT cells (OVCA‐429, OVCA420, HeyA8). Venn diagram illustrating the overlap and unique lipid species detected across (D) TP53 mutant‐EVs and TP53‐WT‐EVs. (E) TP53 mutant cells and TP53‐WT cells. Volcano plot (F) comparing lipidomic profile of EVs and cells from high‐grade serous ovarian cancer (OVCAR‐3, OV‐90, CAOV‐3, PEO1 and PEO4) (G) comparing lipidomic profile of EVs and cells from controls (HOSE 17.1, Met5A and HEK293T). Venn diagram illustrating the overlap and unique lipid species detected across (H) HGSOC‐EV and Control‐EV (I) HGSOC‐cell and control‐cell. The significantly altered lipid species were filtered using adjusted *P* value < 0.05,with LogFC set to ≤ −1 for EVs and ≥ +1 for cells.

By contrast, TP53‐mutant cells had enrichment of 23 lipid species relative to wild type cells. These included mitochondrial cardiolipins (CL54 : 6, CL 64 : 3, CL 66 : 4, CL86 : 11, CL 88 : 16), signaling phospholipids (PA 34 : 2, PE 36 : 0, PI 32 : 0, PIP 32 : 1, PIP 34 : 2, PIP3 34 : 2, PS 40 : 3), storage and signaling lipids such as NAPE (50 : 10, 54 : 12), MADAG (44 : 1, 58 : 8, 60 : 8), MGMG (42 : 12, 48 : 8). Additional lipid species exclusively enriched in TP53 mutant cells included DGPP 30 : 1, GlcdE 16 : 2, TAG 46 : 4, OAHFA 18 : 2/30 : 2 (Fig. 4E). This suggests that TP53 mutations are associated with a broad reprogramming of lipid metabolism, showing the enrichment of storage and signaling lipids such as TAGs, NAPEs and LPS in EVs while the TP53 mutant cells themselves favored mitochondrial resident lipids (cardiolipins), signaling phospholipids (PIP, PIP2, PIP3) and structural or bioactive intermediates (PA, PS, PE). These alterations in TP53 mutant cells might increasingly rely on mitochondrial and phospholipid rewiring to support bioenergetics, survival and oncogenic pathways.

A similar pattern was observed when comparing high‐grade serous ovarian cancer cell lines (OVCAR‐3, OV‐90, CAOV‐3, PEO1 and PEO4) with control non‐cancer cell lines (HOSE 17.1, Met5A and HEK293T) (Fig. 4F,G) (Tables S7.1, S7.2). HGSOC‐derived EVs showed enrichment of TAGs (38 : 2,58 : 12, 58 : 10), NAPEs (34 : 3, 44 : 5, 48 : 7), ceramides (Cer 34 : 2, CerP 26 : 0) and LPS 30 : 0 in EVs (Fig. 4H). In contrast, cardiolipins, phospholipids, sphingomyelins, MADAG, MMPE and DMPE were abundant in HGSOC cells (Fig. 4I). This is consistent with the fact that TP53 mutations, which are associated with high‐grade serous properties, also appear to shape these distinct lipidomic patterns and EV composition during ovarian cancer progression.

### 3.5 Chemoresistant cell lines‐derived EVs are enriched in lipid species linked to membrane  negative intrinsic curvature

We further investigated the lipid alterations in chemoresistance by comparing cisplatin‐resistant (PEO4) and sensitive cell line (PEO1), workflow described in Fig. 5A. Differential expression analysis (Fig. 5B,C) (Tables S8.1 and S8.2) highlighted the significant enrichment of 44 individual lipid species in PEO4 derived EVs relative to PEO1 derived EVs such as TAG (36 : 1, 38 : 3, 40 : 0, 40 : 2, 44 : 2, 44 : 3, 44 : 4, 44 : 5, 46 : 2, 54 : 9, 56 : 3, 56 : 7, 58 : 7, 60 : 12, 60 : 7), ceramides (Cer 34 : 2, CerP 26 : 0), NAPE (42 : 2, 44 : 5, 48 : 7, 52 : 1, 52 : 10, 52 : 5, 52 : 9, 54 : 11), MADAG (40 : 1, 42 : 1, 42 : 2, 44 : 6, 46 : 7, 50 : 7, 60 : 6), CL (60 : 6, 62 : 3), DGDG (34 : 5, 40 : 7), GlcdE (20 : 5, 26 : 1). Along with this, individual lipid species such as DAG 38 : 2, DGMG 20 : 5, GM2 34 : 1, LPG 30 : 2, PA 24 : 4 and PIP3 38 : 1 were enriched in PEO4 cisplatin resistant EVs (Fig. 5D).

**Fig. 5:**
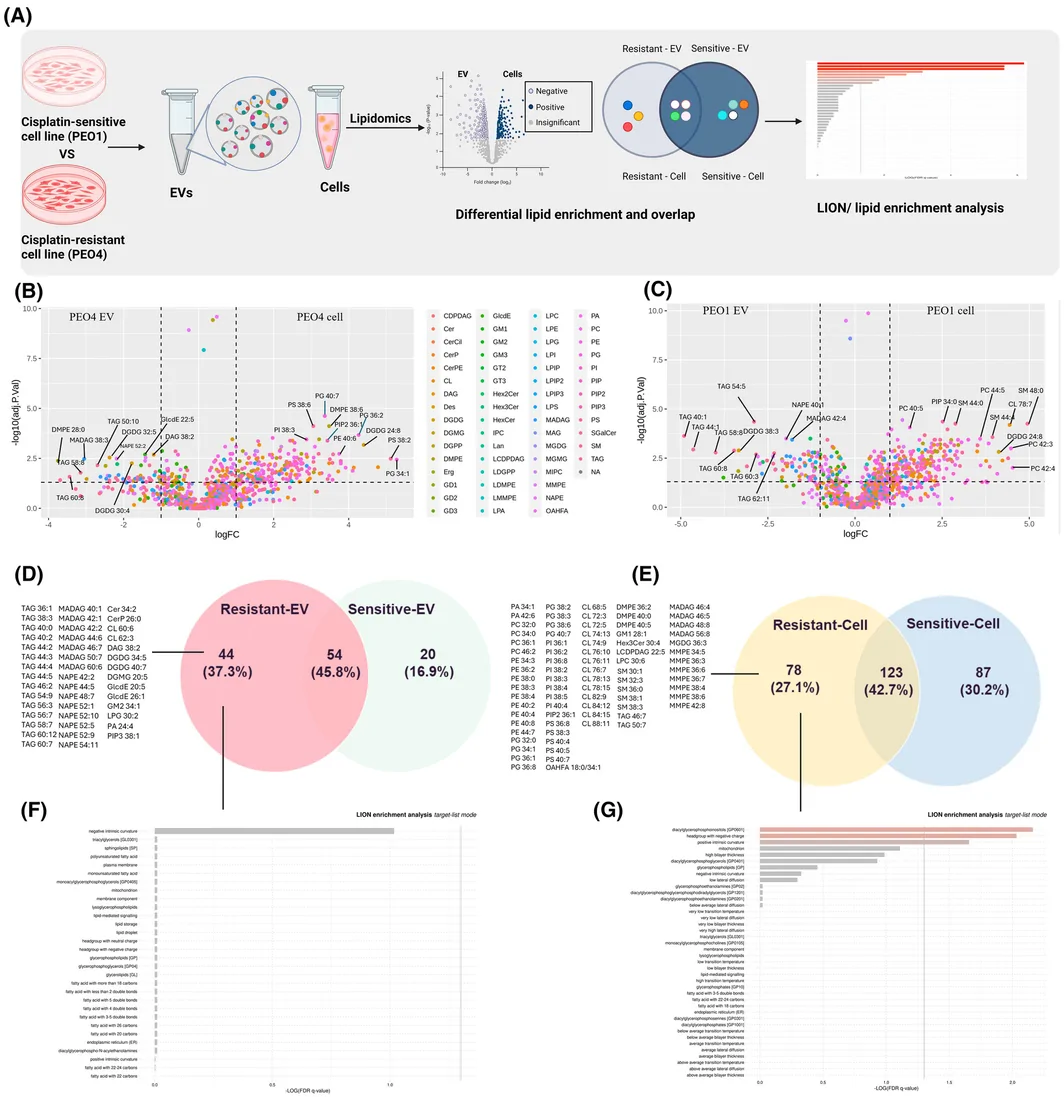
Comparative lipidomic analysis of cisplatin‐sensitive (PEO1) and cisplatin‐resistant (PEO4) cell line and corresponding EVs. (A) Workflow showing differential lipid profiling using LipidSuite, Venn diagram and lipid enrichment analysis. Created in BioRender.com/ga0bwyw. Volcano plot comparing lipidomic profile of (B) cisplatin‐resistant PEO4 EVs vs PEO4 cells (C) cisplatin‐sensitive PEO1 EVs vs PEO1 cells. Venn diagram illustrates the overlap and unique lipid species detected across (D) resistant EVs and sensitive‐EVs (E) resistant cell and sensitive cell. The significantly altered lipid species were filtered using an adjusted *P* value < 0.05, with LogFC ≤ −1 for EVs and ≥ +1 for cells. Lipid ontology enrichment analysis showing the functional role of (F) unique resistant EV lipids (G) unique resistant cell lipids.

In contrast, PEO4 cells had enrichment of 78 lipid species included CL (68 : 5, 72 : 3, 72 : 5, 74 : 13, 74 : 9, 76 : 10, 76 : 11, 76 : 7, 78 : 13, 78 : 15, 82 : 9, 84 : 12, 84 : 15, 88 : 11), DMPE (36 : 2, 40 : 0, 40 : 5), MADAG (46 : 4, 46 : 5, 48 : 8, 56 : 8), MMPE (34 : 5, 36 : 3, 36 : 6, 36 : 7, 38 : 4, 38 : 6, 42 : 8), PA (34 : 1, 42 : 6), PC (32 : 0, 34 : 0, 36 : 1, 46 : 2), PE (34 : 3, 36 : 2, 38 : 0, 38 : 3, 38 : 4, 40 : 2, 40 : 4, 40 : 8, 44 : 7), PG (32 : 0, 34 : 1, 36 : 1, 36 : 8, 38 : 2, 38 : 3, 38 : 6, 40 : 7), PI (36 : 1, 36 : 2, 36 : 8, 38 : 2, 38 : 3, 38 : 4, 38 : 5, 40 : 4), PS (36 : 8, 38 : 3, 40 : 4, 40 : 5, 40 : 7), SM (30 : 1, 32 : 3, 36 : 0, 38 : 1, 38 : 3), TAG (46 : 7, 50 : 7), GM1 28 : 1, Hex3Cer 30 : 4, LCDPDAG 22 : 5, LPC 30 : 6, MGDG 36 : 3, OAHFA 18 : 0/34 : 1 (Fig. 5E).

Functional enrichment analysis by LION web revealed that lipids enriched in resistant EVs were associated with intrinsic membrane curvature and accumulation of storage lipids, whereas lipids enriched in resistant cells showed an association with maintaining membrane thickness and regulation of phosphoinositols (Fig. 5F,G). These findings suggest the divergent adaptations in EVs compared to their parental cells during the development of chemotherapeutic (cisplatin) resistance.

### 3.6 Differential lipid group distribution in cells and EVs associated with ovarian cancer properties

To summarize overall lipidomic changes in ovarian cancer cells and released EVs, significantly altered lipid species were grouped into lipid classes, and their relative proportions (% of total significant lipids) were combined to generate a stacked bar plot depicting lipid enrichment in EV associated with distinct ovarian cancer characteristics such as chemoresistance, TP53 mutation and high‐grade serous cancer phenotype (Fig. 6A). EVs derived from TP53 mutant cells exhibited high proportions of NAPE, MADAG and TAG at 28.57% each, along with 14.29% of LPS. EVs from the HGSOC cells showed balanced enrichment of Cer, CerP, LPS, GlcdE at 10% each with TAG and NAPE representing 30% each. Lipid profiles from chemoresistant EVs were more diverse, dominated by TAG (34.09%), NAPE (18.18%), MADAG (15.91%), DGDG, GlcdE, CL (4.55%) along with minor contributions of 2.27% each of DAG, DGMG, GM2, LPG, PA, PIP3, Cer and CerP each (Fig. 6A).

**Fig. 6:**
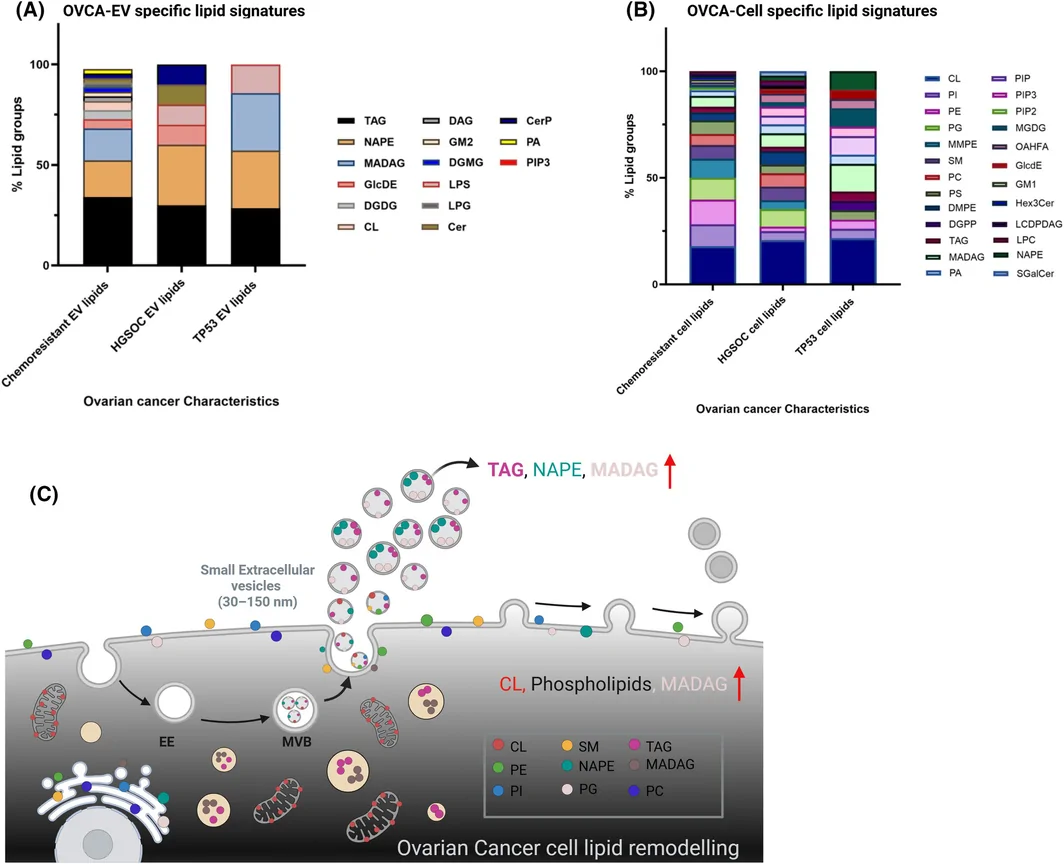
Lipid group composition of EVs and OVCA cells associated with chemoresistance, high‐grade serous phenotype and gene mutation. Stacked bar plots represent the percentage contribution of each lipid group in (A) EVs (B) OVCA‐cells characterized by chemoresistance (left), HGSOC phenotype (middle) and TP53 mutation (right). Lipid species were identified by differential analysis comparing ovarian cancer cells and EV lipidome and those with ≥ 1‐fold change (for cells), ≤ −1 (for EVs) and adjusted *P*‐value < 0.05 were included. Individual lipid species were grouped into broad lipid classes such as TAG, NAPE, MADAG, GlcdE, etc., as shown (C) Schematic illustrating the reciprocal lipid remodeling between parental ovarian cancer cells and their secreted EVs. Created in BioRender.com/tt4cwwm.

In contrast, TP53 mutated cell lines demonstrated enrichment of CL (21.73%), MADAG (13.04%), MGDG, NAPE, PIP (8.69%) along with lower proportion of DGPP, GlcdE, OAHFA, PA, PE, PI, PIP3, PS and TAG (4.34% each) relative to TP53 WT cells. The HGSOC cells retained the enrichment of lipid species CL (20.83%), PG (8.33%), DMPE, MADAG, PC, SM (6.25% each) along with MMPE, OAHFA, PA, PI, PIP, PIP3, PS (4.16% each), GlcdE, LCDPDAG, LPC, MGDG, NAPE, PE, SGalCer, TAG (2.08%) compared to controls. The cisplatin chemoresistant cell (PEO4) was enriched in CL (17.94%), PE (11.53%), PG and PI (10.25% each), MMPE (8.97%), PS, SM (6.41%), PC and MADAG (5.12%), DMPE (3.84%), TAG and PA (2.56%) and trace amount of 1.28% of TAG, PA, OAHFA, MGDG, PIP2, LCDPDAG, GM1, Hex3Cer and LPC compared to their chemosensitive PEO1 counterpart (Fig. 6B).

Despite variability across TP53 status, chemoresistance, and HGSOC phenotypes, EV lipidomes consistently exhibited enrichment of TAG, NAPE, and MADAG, suggesting their preferential packaging into vesicles. Conversely, parental cells were predominantly enriched in cardiolipins, phospholipids, and MADAG, demonstrating a clear divergence between EV and cellular lipid compositions (Fig. 6C).

## 4 Discussion

Ovarian cancer cells rely heavily on lipids within the tumor microenvironment (TME) as an energy source to sustain rapid proliferation and membrane biogenesis, and this lipid dependency is now recognized as a hallmark of malignancy‐associated metabolic reprogramming [40, 41]. This dysregulation manifests both within the tumor and throughout its surrounding TME [42], consistent with the preferential metastasis of ovarian cancer cells to lipid‐rich niches such as the intestinal lining and peritoneal cavity [3, 43]. Understanding the interplay between ovarian cancer phenotypes and lipid metabolism is therefore central to elucidating mechanisms of progression, survival, and therapeutic resistance. Malignant ascites, rich in tumor and stromal components, constitutes a lipid‐rich niche and serves as a proximal reservoir of tumor‐derived EV biomarkers [44, 45], but ascites does not form in all ovarian cancer patients, and in advanced stages of the disease, limiting its utility as a potential liquid biopsy source. Plasma is more accessible but dilutes tumor‐derived EV signals within the systemic circulation. Lipid biomarkers that are detectable in both ascites and blood, and that also trace back to the tumor cells themselves, would therefore combine diagnostic sensitivity with practical accessibility. Our comparative lipidomic profiling of ascites, plasma, and ovarian cancer cell lines and their EVs was designed to identify such a panel.

Interestingly, a small core of lipid species was shared across all four EV sources (14 species, 2%), while the largest pairwise overlap outside the cell‐line‐derived EVs was between plasma‐ and ascites‐derived EVs (13 species), candidates that are, by definition, detectable in both clinically accessible biofluids and therefore of particular interest for biomarker development. Among the EV‐enriched lipid classes, the consistent enrichment of triglycerides (TAG), N‐acyl phosphatidylethanolamines (NAPE), and monoalkyl‐diacylglycerols (MADAG) in EVs from HGSOC and cisplatin‐resistant cells stood out. TAG is both a dynamic energy source and a survival factor for normal and cancerous cells alike [46, 47, 48]; excess cholesterol, phospholipids, and TAG within tumors are either exported into circulation or stored as intracellular lipid droplets as a strategy to manage lipidomic overload [49], and TAG‐enriched EVs have similarly been reported in malignant pleural effusions [50] and hypoxic prostate cancer cells [22], suggesting broad oncogenic relevance. Such EVs likely remodel the TME by delivering bioactive lipids and lipogenic enzymes to recipient cells : ovarian tumor‐derived EVs stimulate lipid release from omental adipocytes and drive CD36‐mediated lipid uptake in macrophages, promoting a pro‐metastatic, senescent macrophage phenotype enriched in TAG, DAG, free fatty acids, and ceramides [51].

Our finding of TAG and MADAG enrichment specifically in OVCA‐derived EVs is consistent with a potential mechanistic link to this macrophage reprogramming and omental metastatic niche formation. Given its structural similarity to DAG, MADAG may share DAG's role in activating protein kinase C and related oncogenic signaling pathways relevant to angiogenesis and metastasis [34], and NAPE enrichment in OVCA‐EVs may reflect a potential signaling role in tumor progression [52, 53]; however, direct functional evidence for either lipid class in cancer EV biology remains limited. In contrast to their EVs, parental ovarian cancer cells were prominently enriched in mitochondrial and membrane lipids, including cardiolipins (CL), phosphatidylinositols (PI), phosphatidylethanolamines (PE), and phosphatidylglycerols (PG), pointing to distinct but complementary roles for EVs and cells in metabolic remodeling and intercellular communication. CLs are anionic phospholipids found exclusively at the mitochondrial membrane, where they maintain mitochondrial function and integrity [54, 55, 56, 57, 58] and support bioenergetics, apoptosis, mitophagy, and dynamics, processes tied to cancer aggressiveness, including migration, invasion, and treatment resistance [59]. CL abundance is tumor‐type specific, elevated in prostate [60] and thyroid cancer [61] but reduced in hepatocellular carcinoma [62]. Consistent with this, cisplatin‐resistant PEO4 cells showed enrichment of 14 cardiolipin species relative to sensitive PEO1 cells, in line with reports that PEO4 cells exhibit increased mitochondrial activity and ROS production correlating with higher mitochondrial numbers [63], linking CL enrichment to mitochondrial adaptation in chemoresistance. Beyond CLs, enrichment of PC, PI, PA, and PS has been associated with prostate cancer aggressiveness, and the consistent increase in PG and PA we observed in ovarian cancer cells aligns with heightened proliferation and phospholipid biosynthesis demand [64]. PA, in particular serves as a precursor for diverse glycerophospholipids via two branching pathways, conversion to DAG, which generates PC and PE, or to CDP‐DAG, which generates PG, PI, and PS, and acts as a lipid second messenger regulating cell cycle progression, growth, and survival [65].

Several limitations of this study should be noted. Ascites samples were available from a comparatively small number of patients, and plasma and ascites were not collected from the same individuals, precluding direct within‐patient comparison of systemic and tumor‐proximal EV lipid profiles. Cell lines, while a tractable model for nominating candidate EV lipid signatures, do not fully recapitulate the complexity of the *in vivo* tumor microenvironment, and findings derived from cell line comparisons, including the TP53‐mutation and cisplatin‐resistance analyses, should be interpreted as hypothesis‐generating rather than validated in patients. Given the substantial molar excess of lipoproteins relative to EVs in plasma, complete separation of EVs from lipoproteins by size‐exclusion chromatography is not achievable, and a subset of EVs may associate directly with lipoproteins in circulation; the enrichment of TAG‐, NAPE‐, and MADAG‐related species should therefore be interpreted with particular caution in plasma, where co‐isolated lipoprotein particles are more abundant than in ascites. To mitigate this, candidate lipid markers were prioritized based on reproducible enrichment relative to matched control EV fractions across both the discovery and validation cohorts, and we highlight species showing consistent enrichment across independent patient sets as the most robust candidates. The lipid species panels were generated from Venn diagrams of EV lipids enriched relative to their cells of origin. We prioritized a set of lipids unique to ascites EVs, shared between OVCA cell_EVs and control cell EVs, shared among OVCA cell_EVs, control_cell EVs, and OVCA plasma_EVs, and unique to OVCA plasma_EVs. These candidates were then refined using SHAP to select the most informative 20 lipid species panel within each classifier and were tested in an independent validation cohort. Across plasma and matched EV classifiers (control vs OVCA, benign vs OVCA, control_benign vs OVCA), the resulting panels comprised source‐specific combinations of lipid species from ascites‐derived EVs, shared cell‐derived EVs and OVCA_plasma‐derived EVs, included ceramides, cardiolipins, diacylglycerols, phospholipids, hexosylceramides, cholesteryl esters, and triglycerides.

Finally, because plasma and ascites contain heterogeneous EV populations derived from immune, stromal, and mesothelial cells in addition to tumor cells, and because our workflow nominated candidate lipids from cell‐line‐derived EVs rather than through direct tumor‐specific enrichment in patient samples, we describe the identified lipids as ovarian cancer‐associated rather than of confirmed tumor‐exclusive origin. The precise mechanisms governing EV lipid sorting, and how specific lipid alterations confer survival advantage within the lipid‐rich TME, remain open questions; future work should also examine how TP53 mutation status and lipid metabolic reprogramming jointly influence chemotherapy response in patients.

## 5 Conclusion

This study provides an extensive multicompartment lipidomic characterization of ascites, plasma, and ovarian cancer cell lines alongside their matched EVs, highlighting lipidomic adaptations that accompany the development of invasive and chemoresistant phenotypes. The selective export of energy‐storage and signaling lipids via EVs illustrates a tumor strategy for modulating both intracellular lipid homeostasis and the local microenvironment, pointing to potential avenues for targeted metabolic therapies. These findings identify EV‐associated lipid species with candidate biomarker relevance and provide a foundation for future investigation into EV biogenesis and lipid rewiring in ovarian cancer progression.

## Conflict of interest

The authors declare no conflicts of interest.

## Author contributions

SR contributed to conceptualization; formal analysis; investigation; visualization; writing–original draft; writing–review and editing. DG contributed to formal analysis, methodology, visualization. AL contributed to formal analysis, methodology, visualization. KS‐R contributed to investigation, and methodology. S. Reed and ER contributed to lipidomic analysis, investigation, and methodology. RL and NC contributed to clinical samples analysis, investigation, and methodology. KF contributed to clinical samples analysis, investigation, and methodology. CS contributed to conceptualization, funding acquisition, investigation, project administration, supervision, writing–original draft, writing–review and editing.

## Funding

C.S. is supported by The Medical Research Future Fund (MRF1199984), National Health and Medical Research Council (NHMRC 1195451), The Donald & Joan Wilson Foundation Ltd. (2020000323), and the Ovarian Cancer Research Foundation (OCRF, 2018001167).

## Supporting information


**Fig. S1.** Immunoblot analysis of size‐exclusion chromatography (SEC) fractions.
**Fig. S2.** Concentration and mode particle size of EVs across ovarian cancer cell lines and plasma samples.
**Table S1.** Summary of key characteristics of ovarian cancer and control cell lines.

**Table S2.** Statistical comparison of lipid species between ovarian cancer cell lines (*n* = 10) and matched EV preparations.

**Table S3.** Statistical comparison of lipid species between control cell lines (*n* = 3) (Control_cell) and matched EV preparations (Control_EV).

**Table S4.** Statistical comparison of lipid species between HGSOC patient plasma (*n* = 46) (OVCA_Plasma) and matched EV preparations (OVCA_EV).

**Table S5.** Statistical comparison of lipid species between HGSOC patient ascites (*n* = 7) and matched EV preparations (Ascites_EV).

**Table S6.1.** Summary of differential lipid species analysis between TP53‐WT ovarian cancer cells and matched EVs.
**Table S6.2.** Summary of differential lipid species analysis between TP53‐mutated ovarian cancer cells and matched EVs.

**Table S7.1.** Summary of differential lipid species analysis between control cells and matched EVs.
**Table S7.2.** Summary of differential lipid species analysis between HGSOC cells and matched EVs.

**Table S8.1.** Summary of differential lipid species analysis between cisplatin‐ sensitive (PEO1) and matched EVs.
**Table S8.2.** Summary of differential lipid species analysis between cisplatin‐ resistant (PEO4) and matched EVs.

## Data Availability

All data supporting the findings of this study are available within the article and the supplemental information, or from the corresponding author upon reasonable request.
