# Supplementary material for: Extracellular vesicle lipidomics liquid biopsy reveals systemic and tumor microenvironment metabolic reprogramming in ovarian cancer
Source: Mol Oncol. 2026 Oct 7:10.1002/1878-0261.70327. Online ahead of print. doi: 10.1002/1878-0261.70327 (PMC13643885; doi:10.1002/1878-0261.70327)
Supplement: Supplementary file 1 [file MOL2-9999-0-s008.docx]

**Supplementary materials:**

**Figure S1:**

**Figure S1:** Immunoblot analysis of SEC fractions. Immunoblotting was performed on SEC fractions F1-F3 to assess EV-enriched and non-EV associated proteins. EV transmembrane protein; CD9 and cytosolic protein; Flotillin-1 were detected in fraction 1-3, whereas albumin was detected in later fraction (F4-F13), indicating successful separation of EV-containing fractions.

**Figure S2:**

**Figure S2:** Concentration and mode particle size of EVs across ovarian cancer cell lines and plasma samples (A) Concentration of EVs (Particle/mL) analyzed across 13 cell lines including non-cancer and ovarian cancer cell lines. (B) Mode particle size(nm) of EVs measured in these cell lines (C) Concentration (Particle/mL) and mode size (nm) of EVs in plasma samples from control, benign, and OVCA patients. Data are presented as mean ± SEM, statistical analysis was performed using ordinary one way-ANOVA.

**Table S1: Characteristics of ovarian cancer and control cell lines**

| **Cell lines** | **TP53 status** | **Histological types** | **Origin** | **Primary/metastasis** | **References** |
| --- | --- | --- | --- | --- | --- |
| TOV112D | Mutated | Ovarian endometrioid adenocarcinoma | Ovary endometrioid-stage IIIC-grade 3 | Primary | [1-4] |
| OVCAR-3 | Mutated | Adenocarcinoma, high grade serous | Ascites | Metastasis | [3-6] |
| OV-90 | Mutated (Tumorigenic) | Adenocarcinoma, high grade serous | Ascites-stage IIIC-grade 3 | Metastasis | [4, 7, 8] |
| HeyA8 | WT(Tumorigenic) | High grade ovarian serous Adenocarcinoma, | Tumor-serous | Metastasis | [4, 7, 9] |
| SKOV-3 | Null | Ovarian serous Cystadenocarcinoma | Ascites | Metastasis | [7, 8] |
| OVCA-420 | WT(Tumorigenic) | Ovarian serous adenocarcinoma | Ovary | Ovary | [10] |
| OVCA-429 | WT(Tumorigenic) | Ovarian cystadenocarcinoma | Ovary | Primary | [10] |
| CAOV-3 | Mutated | High grade ovarian serous adenocarcinoma | Ovary | Primary | [3, 7, 8, 11] |
| PE01 | Mutated | Ovarian Cystadenocarcinoma (Chemosensitive)-cisplatin | 1^st^ relapse Ascites-stage III | Metastasis | [3, 4, 8, 12] |
| PE04 | Mutated | Ovarian Cystadenocarcinoma (Chemo resistant) -cisplatin | 2^nd^ relapse Ascites | Metastasis | [3, 8, 12] |
| HOSE 17.1 | WT (non-tumorigenic, not mutated) | Human ovarian surface epithelium(non-cancerous) | Ovary | Primary | [13] |
| HEK293T | Mutant (inactivated TP53 function) | Embryonic kidney, epithelial(transformed) | Human embryonic kidney | Primary | [14] |
| Met5a | WT (non-tumorigenic, not mutated) | Human mesothelial (non-cancerous) | Human pleural mesothelial cells | Primary | [15] |

**References**

1.	Freedman, R.S., et al., *Characterization of an ovarian carcinoma cell line.* Cancer, 1978. **42**(5): p. 2352-9.

2.	Provencher, D.M., et al., *Characterization of four novel epithelial ovarian cancer cell lines.* In Vitro Cell Dev Biol Anim, 2000. **36**(6): p. 357-61.

3.	Yee, C., et al., *Three-Dimensional Modelling of Ovarian Cancer: From Cell Lines to Organoids for Discovery and Personalized Medicine.* Front Bioeng Biotechnol, 2022. **10**: p. 836984.

4.	Houshdaran, S., et al., *DNA methylation profiles of ovarian epithelial carcinoma tumors and cell lines.* PLoS One, 2010. **5**(2): p. e9359.

5.	Hamilton, T.C., et al., *Characterization of a human ovarian carcinoma cell line (NIH:OVCAR-3) with androgen and estrogen receptors.* Cancer Res, 1983. **43**(11): p. 5379-89.

6.	De Haven Brandon, A., et al., *Identification of ovarian high-grade serous carcinoma cell lines that show estrogen-sensitive growth as xenografts in immunocompromised mice.* Sci Rep, 2020. **10**(1): p. 10799.

7.	Domcke, S., et al., *Evaluating cell lines as tumour models by comparison of genomic profiles.* Nature Communications, 2013. **4**(1): p. 2126.

8.	Hernandez, L., et al., *Characterization of ovarian cancer cell lines as in vivo models for preclinical studies.* Gynecol Oncol, 2016. **142**(2): p. 332-40.

9.	Buick, R.N., R. Pullano, and J.M. Trent, *Comparative Properties of Five Human Ovarian Adenocarcinoma Cell Lines1.* Cancer Research, 1985. **45**(8): p. 3668-3676.

10.	E, Ó.h., et al., *Mutation or loss of p53 differentially modifies TGFβ action in ovarian cancer.* PLoS One, 2014. **9**(2): p. e89553.

11.	Buick, R.N., R. Pullano, and J.M. Trent, *Comparative properties of five human ovarian adenocarcinoma cell lines.* Cancer Res, 1985. **45**(8): p. 3668-76.

12.	Langdon, S.P., et al., *Characterization and properties of nine human ovarian adenocarcinoma cell lines.* Cancer Res, 1988. **48**(21): p. 6166-72.

13.	Anugraham, M., et al., *Specific glycosylation of membrane proteins in epithelial ovarian cancer cell lines: glycan structures reflect gene expression and DNA methylation status.* Mol Cell Proteomics, 2014. **13**(9): p. 2213-32.

14.	Stepanenko, A.A. and V.V. Dmitrenko, *HEK293 in cell biology and cancer research: phenotype, karyotype, tumorigenicity, and stress-induced genome-phenotype evolution.* Gene, 2015. **569**(2): p. 182-190.

15.	Bioinformatics), C.S.I.o. *MeT-5A (Cellosaurus CVCL_3749; RRID:CVCL_3749)*. 2025; Available from: <https://www.cellosaurus.org/CVCL_3749>.
